# Supplementary material for: Observation and rationalization of nitrogen oxidation enabled only by coupled plasma and catalyst
Source: Nat Commun. 2022 Jan 20;13:402. doi: 10.1038/s41467-021-27912-2 (PMC8776816; doi:10.1038/s41467-021-27912-2)
Supplement: Supplementary file 1 — Supplementary Information [file 41467_2021_27912_MOESM1_ESM.pdf]

Supplementary Information: Observation and rationalization of nitrogen oxidation enabled only by coupled plasma and catalyst

Hanyu Ma,[†] Rakesh K. Sharma,[‡] Stefan Welzel,[‡] Mauritius C.M. van de Sanden,[‡,¶] Mihalis N. Tsampas,[*,‡] and William F. Schneider[*,†,§]

†*Department of Chemical and Biomolecular Engineering, University of Notre Dame, Notre Dame, Indiana 46556, United States*

‡*Dutch Institute for Fundamental Energy Research (DIFFER), De Zaale 20, 5612 AJ, Eindhoven, The Netherlands*

¶*Department of Applied Physics, Eindhoven University of Technology (TU/e), 5600 MB Eindhoven, The Netherlands*

§*Department of Chemistry and Biochemistry, University of Notre Dame, Notre Dame, Indiana 46556, United States*

E-mail: m.tsampas@differ.nl; wschneider@nd.edu

SUPPLEMENTARY METHODS

This file provides additional information of methods, figures and tables.

Equilibrium nitrogen oxide and nitrogen dioxide composition of dry air

Supplementary Fig.1 shows the equilibrium pressures of a gas mixture of 0.78:0.21 N_2:O_2 in a constant volume reactor initially set at 1 bar. The pressures are solved with experimentally measured thermodynamic constants.[1]

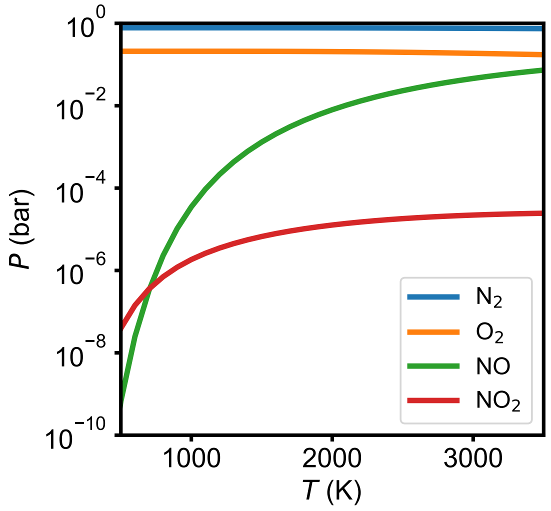

Supplementary Figure 1: Constant volume equilibrium composition of a 0.78:0.21 N_2:O_2 gas mixture initially at 1 bar total pressure.

Afterglow and temperature

Supplementary Fig.2 shows the temperature of the plasma was above 450 K and decreased with the distance from the coil. Without the heating mantle, the temperature dropped to about 320 K at 18 cm downstream from the coil. The Pt catalyst was therefore placed 30 cm away from the coil to minimize the impact of the plasma afterglow on the surface of the catalyst. We always use this distance to ensure the catalyst is not impacted by the visible

region of the plasma afterglow and the active plasma species can still reach the surface of the catalyst.

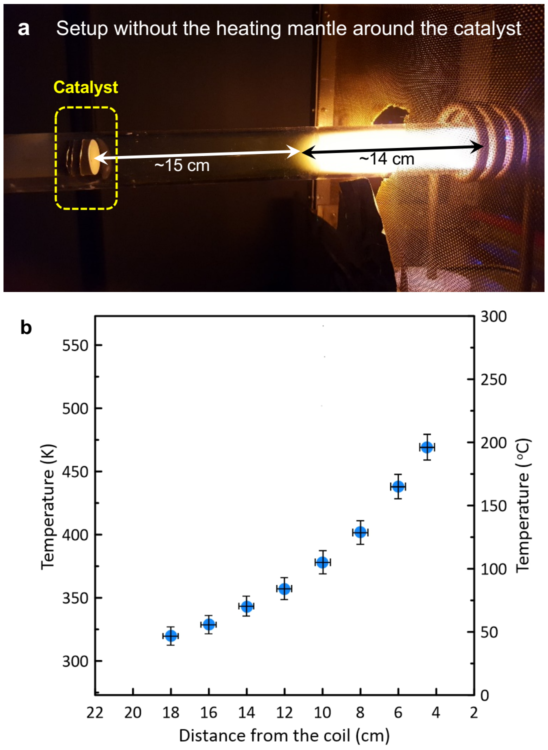

Supplementary Figure 2: (a) Plasma afterglow without the heating mantle. Plasma was on the right and the gas flowed from the right to the left. (b) Temperature as a function of distance from the coil.

XRD and XPS of the catalyst

Supplementary Fig.3a plots the XRD profile of the as-prepared Pt supported on yttria stabilized zirconia. The XRD profile of the catalysts after reactions shows negligible difference from Supplementary Fig.3a, suggesting Pt was not oxidized or nitrified after N_2 oxidation. Supplementary Fig.3b compares the XPS profiles of Pt before and after reaction. These two profiles overlap with each other. We further analyze the XPS profile of Pt. The baseline of the XPS spectrum is fitted with a Shirley algorithm and the two peaks are fitted with a Pseudo-Voigt function. The fitted curve matches the profiles of Pt before and after plasma

catalytic reactions. Two peaks, i.e. Pt $4f_{5/2}$ and Pt $4f_{7/2}$, are observed. These results suggest both the surface and bulk of Pt remain metallic after the reactions in plasma with O_2 mole fractions smaller than 1%. Fig.3c shows Pt was partially oxidized after exposure of 20% O_2 plasma.

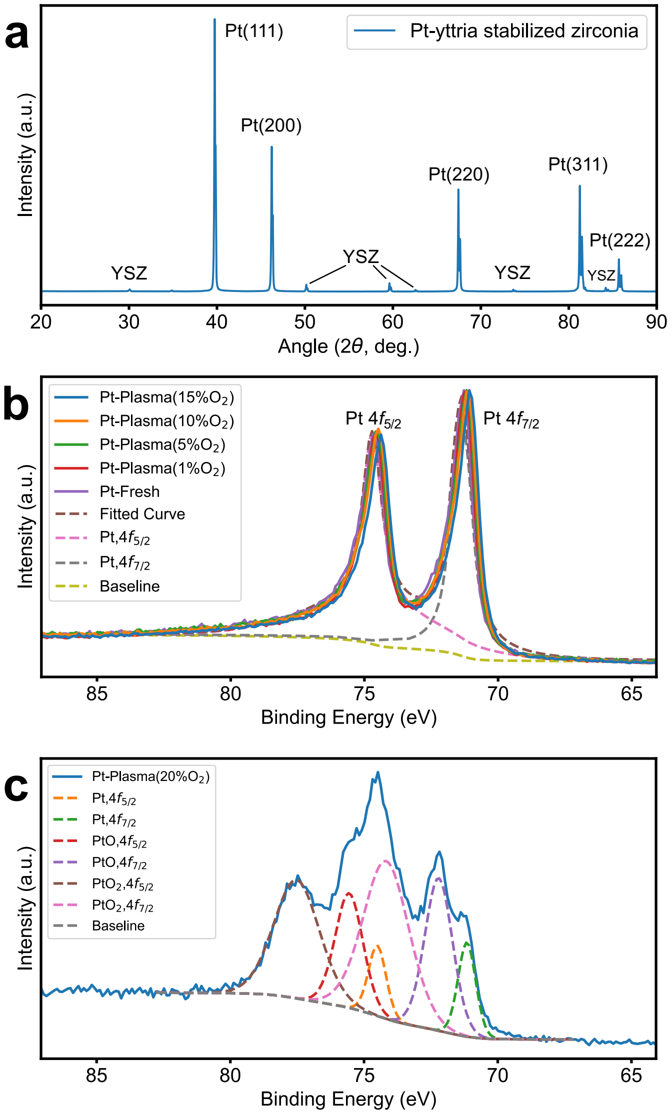

Supplementary Figure 3: (a) X-ray diffraction of porous Pt film supported on yttria stabilized zirconia(YSZ). X-ray photoelectron spectroscopy (XPS) of (b) fresh Pt and Pt after exposure of 1, 5, 10 and 15% O_2 plasma and (c) Pt after exposure of 20% O_2 plasma for 3 h.

Pt surface reaction parameters

We define the rate constants of adsorption, surface and desorption reactions on Pt with transition state theory.[2]

$$k = A\exp\left(-\frac{E_{\mathrm{a}}}{k_{\mathrm{B}}T}\right) \tag{1}$$

$$A = \frac{k_{\mathrm{B}}T}{h}\exp\left(\frac{\Delta S^{\circ}_{\mathrm{TS}}}{k_{\mathrm{B}}}\right) \tag{2}$$

Here, k_{B} is the Boltzmann constant, T is the absolute temperature, h is the Planck constant, $\Delta S^{\circ}_{\mathrm{TS}}$ is the standard entropy difference between the transition state and the initial state, and E_{a} is the energy difference between the transition state and the reactants at infinite separation, as listed in Table 1. For adsorption reactions, we used the entropies of gas molecules from NIST-JANAF thermochemical tables.[1] We assumed the entropies of transition states equal to the summation of the corresponding atomic adsorbate entropies since the diatomic transition states largely lost their molecular identities and resemble the dissociated atoms.[3] The entropies of the atomic adsorbates over Pt(211) are taken from our previous calculations with harmonic oscillator approximation.[4,5] We assume surface reactions are entropy conservative and $A = \frac{k_{\mathrm{B}}T}{h}$.

Supplementary Table 1: Reaction and activation energies of N_2 oxidation on Pt(111) and Pt(211). (*) represents a binding site. The barriers are the energy differences between the transition states and the initial states at infinite separation.[6]

**Reactions on Pt Surfaces**	**(211)**		**(111)**	
	ΔE	E_{a}	ΔE	E_{a}
(1) $N_2 + 2^* \longleftrightarrow 2N^*$	1.35	2.55	1.45	3.90
(2) $O_2 + 2^* \longleftrightarrow 2O^*$	-2.09	0.17	-1.49	0.62
(3) $N + {}^* \longleftrightarrow N^*$	-4.26	0.00	-4.21	0.00
(4) $O + {}^* \longleftrightarrow O^*$	-3.66	0.00	-3.36	0.00
(5) $N^* + O^* \longleftrightarrow NO^* + {}^*$	-0.61	1.39	-0.46	2.09
(6) $NO^* \longleftrightarrow NO + {}^*$	1.89	1.89	1.39	1.39

Alternatively, the prefactor A of adsorption reactions can be estimated using the Hertz-Knudsen equation derived from collision theory.[7] We calculated the prefactors with the

Hertz-Knudsen equation to compare the difference between the two approaches:

$$A = \frac{\sigma S}{\sqrt{2\pi m k_{\mathrm{B}} T}} \times 10^5 \mathrm{Pa} \tag{3}$$

Here, σ is a sticking coefficient, S is the area of one free site, m is the molecular weight of a gas species. We take sticking coefficient here to be unity, as an upper limit on adsorption rates.[8] Table 2 shows that prefactors estimated from Equation 2 are three to four fold smaller than those from Equation 3. In the microkinetic models, we use prefactors estimated from the transition state theory as lower limits, which are equivalent to the prefactors estimated with collision theory using small sticking coefficients.

Supplementary Table 2: Prefactors of adsorption reactions estimated with Equation 2 and Equation 3.

**Adsorption reactions**	Equation 2 ($10^4 s^{-1}$)	Equation 3 ($10^8 s^{-1}$)
(1) N_2 +2* $\longleftrightarrow$ 2N*	8.2	1.2
(2) O_2 +2* $\longleftrightarrow$ 2O*	10	1.1
(3) N + * $\longleftrightarrow$ N*	56	1.6
(4) O + * $\longleftrightarrow$ O*	58	1.5
(5) NO + * $\longleftrightarrow$ NO*	2.0	1.1

Vibrational excitation of molecular nitrogen and oxygen

We estimate the normalized density of each vibrational excited state using with the Treanor equation.[9,10] We include the first ten vibrational excited states because of the depopulation of highly excited levels. Here, $\hbar$ is the reduced Planck's constant, ω is the vibrational frequency and x_e is the anharmonicity coefficient. ω of N_2 and O_2 are 2358.57 and 1580.16 cm^{-1}, respectively. $x_e\omega$ are 14.324 and 11.951 cm^{-1}, respectively.[11]

$$p_v(v, T_{\mathrm{vib}}, T_{\mathrm{gas}}) = \frac{\exp\left(-\frac{\hbar\omega v}{T_{\mathrm{vib}}} + \frac{\hbar x_e \omega v^2}{T_{\mathrm{gas}}}\right)}{\sum_{v=0}^{10} \exp\left(-\frac{\hbar\omega v}{T_{\mathrm{vib}}} + \frac{\hbar x_e \omega v^2}{T_{\mathrm{gas}}}\right)} \tag{4}$$

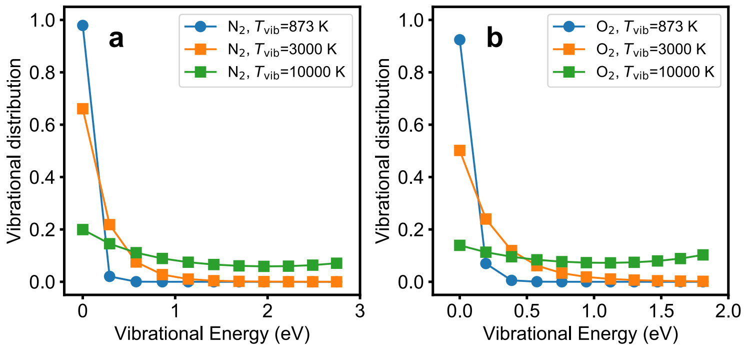

Supplementary Figure 4: Treanor vibrational distribution functions for (a) N_2 and (b) O_2 at $T_{\text{vib}} = 873$, 3000 and 10 000 K and $T_{\text{gas}} = 873$ K.

Vibrational excitations of N_2 and O_2 reduce the activation barriers involving these two species.[10,12] We therefore write the rate constant of each vibrational state, v, of the Zeldovich reactions and the dissociative adsorption of N_2 and O_2 on Pt as:

$$k_v = A\exp\left(-\frac{E_{\text{a}} - \alpha E_v}{k_{\text{B}}T}\right) \tag{5}$$

Here, A is pre-exponential factor defined in the previous sections. E_{a} is activation energy of the ground vibrational state. E_v is the vibrational energy. α is the efficiency of vibrational excitation energy in overcoming the activation barrier. α is estimated with the Fridman-Macheret α-model, $\alpha = \frac{E_{\text{a}}^{(f)}}{E_{\text{a}}^{(f)} + E_{\text{a}}^{(b)}}$, where $E_{\text{a}}^{(f)}$ and $E_{\text{a}}^{(b)}$ are the activation barriers for the forward and backward reactions, respectively.[10] If $E_{\text{a}} < \alpha E_v$, $k_v = A$. The overall k for the adsorption of N_2 or O_2 is calculated with the summation of the rates at different vibrational states:

$$k_{\text{ads}} = \sum_{v=0}^{10} p_v k_v \tag{6}$$

Mean-field microkinetic model

The surface coverages of adsorbates were solved with a mean-field microkinetic model assuming surface coverages reach steady-state at fixed pressures of gaseous species.[13] The coverage

(θ_i) of surface species i is written as a differential equation:

$$\frac{\partial \theta_i}{\partial t} = \sum_j c_{ij} r_j \tag{7}$$

and

$$r_j = k_{+j} \prod_i \theta_i^{-c_{ij}} \prod_i P_i^{-c_{ij}} - k_{-j} \prod_i \theta_i^{c_{ij}} \prod_i P_i^{c_{ij}} \tag{8}$$

where r_j is the net rate of surface reaction j and c_{ij} is the stoichiometric coefficient of species i in reaction j. $c_{ij} < 0$ when i is a reactant, $c_{ij} > 0$ when i is a product and $c_{ij} = 0$ when i is not involved in j. $+j$ and $-j$ represent the forward and backward reactions of j, respectively. k is the rate constant. In Equation 8, θ_i and P_i are the coverage of surface species and the pressure of gaseous species i involved in reaction j, respectively. The first term on the right side of Equation 8 includes species in the forward reaction $+j$. The second term includes species in the backward reaction $-j$. The total coverage on the surface is conserved:

$$\theta_* + \sum_i \theta_i = 1 \tag{9}$$

where θ_* is the coverage of free sites. We solved Equation 7 of all surface species at steady state, where $\frac{\partial \theta_i}{\partial t} = 0$. We used a method that automatically switches between nonstiff (Adams) and stiff (BDF) solvers, as implemented in scipy.integrate.odeint in Python. The steady-state coverages were also solved from a system of algebraic equations when $\sum_j c_{ij} r_j = 0$. Here, the coverages solved from Equation 7 were used as initial guesses to confirm the steady-state coverages. Newton's method, as implemented in mpmath.findroot in Python, was used. Supplementary Fig. 5 shows the steady-state coverages over Pt(211) at different plasma parametric conditions.

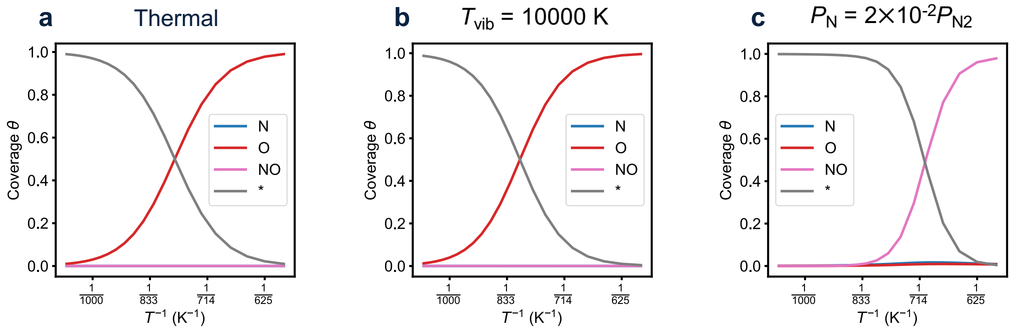

Supplementary Figure 5: Surface coverages of N_2 oxidation on Pt(211) at 873 K. (a) Thermal catalysis, (b) $T_{\text{vib}} = 10\,000$ K and (c) $P_{\text{N}} = 2 \times 10^{-2} P_{\text{N}_2}$. The partial pressures of N_2 and O_2 are 4.995 and 0.005 mbar, respectively.

Degree of rate control

We identify the elementary steps most responsible for the overall NO production rates by performing degree of rate control at different conditions.[14] In Eqn. 10, we calculate rate sensitivity to specific reactions, $X_{\text{RC},i}$, by changing the forward (k_{+i}) and backward (k_{-i}) rate constants of step i simultaneously by 1% while holding constant the rate constants of other steps, k_j, and monitoring the change of the overall rate (r) with respect to the reference rate, r_0.

$$X_{\text{RC},i} = \frac{k_i}{r}\left(\frac{\partial r}{\partial k_i}\right)_{k_{j\neq i},K_i} \approx \frac{k_{i,0}}{r_0}\cdot\frac{r-r_0}{k_i-k_{i,0}} = \frac{r-r_0}{0.01r_0} \tag{10}$$

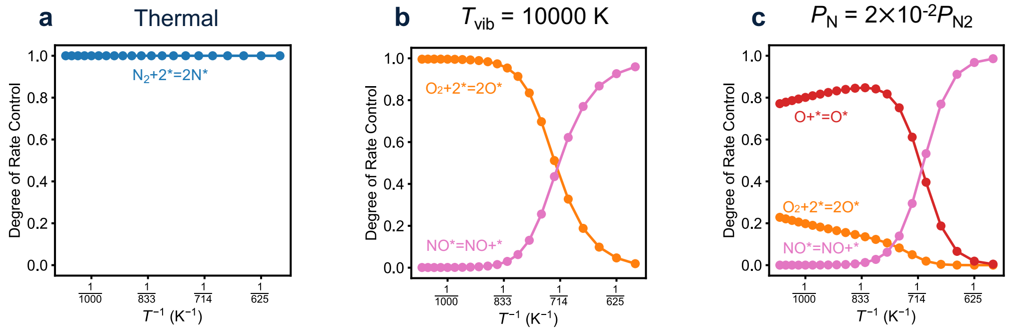

Supplementary Figure 6: Degree of rate control of N_2 oxidation on Pt(211) at 873 K. (a) Thermal catalysis (b) $T_{\text{vib}} = 10\,000$ K and (c) $P_{\text{N}} = 2 \times 10^{-2} P_{\text{N}_2}$. The partial pressures of N_2 and O_2 are 4.995 and 0.005 mbar, respectively.

TOF and NO production on Pt(111)

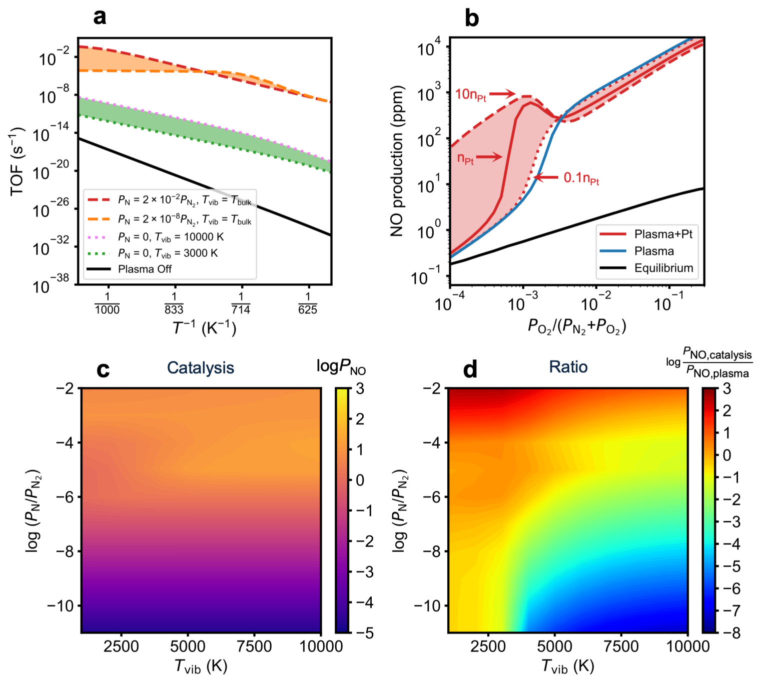

Supplementary Figure 7: (a) Reaction rates against reciprocal temperature on Pt(111) at different vibtational temperature and P_{N}. Pressures of N_2 and O_2 are 4.995 and 0.005 mbar, respectively. (b) Microkinetic prediction of NO production as a function of O_2 pressure in both plasma and plasma catalytic systems with combination of three integral reactors (Figure 5b). $P_{\mathrm{N}} = 2 \times 10^{-3} P_{\mathrm{N_2}}$ and $T_{\mathrm{vib}} = 6000\,\mathrm{K}$ at 873 K. The Pt(111) sites, $\mathrm{n_{Pt}}$, are varied from 23 to 2300 nmol in the plasma catalytic model. (c) Outlet NO pressures (ppm) of plasma reactions against T_{vib} and P_{N} at 873 K in reactors shown in Figure 3b, where the inlet pressures of N_2 and O_2 are 4.995 and 0.005 bar, respectively. The residence times is 6.4 µs. The number of Pt(111) sites is 230 nmol. (d) The ratio of NO produced with plasma catalytic and plasma reactions.

Kinetic parameters of Zeldovich reactions

We use experimentally measured forward rate constants for reactions in the Zeldovich mechanism.[15] The rate constants are in Arrhenius form, $k = A\exp\left(-\frac{E_{\mathrm{a}}}{k_{\mathrm{B}}T}\right)$. We calculate the backward rate constants with standard free energies to enforce thermal consistency.[1] The calculated backward rate constants are in agreement with experimental measurements.[16,17]

Supplementary Table 3: Forward rate constants, standard enthalpy and standard entropy of reactions in the Zeldovich mechanism. T represent gas temperature.

**Reactions**	A (cm^{3}s^{-1})	E_a(eV)	$\Delta H°$(eV)	$\Delta S°$(meV T^{-1})
$N_2 + O \longleftrightarrow NO + N$	3.0×10^{-10}	3.31	3.26	0.123
$O_2 + N \longleftrightarrow NO + O$	$3.2\times10^{-12}(T/300)$	0.27	-1.38	0.139

NO production in integral reactor models

To model NO product concentration, we assume the flow is well-mixed and thus constant throughout the reactor volume, commonly termed a continuously stirred tank reactor (CSTR). Test calculations modeling the system in plug flow yield similar results (Section sec:PFR). In this reactor, the pressure of gaseous species i is governed by Equation 11.

$$V\frac{\partial P_i}{\partial t} = v(P_{i,0} - P_i) + RTVr_{\text{plasma}} + RTSr_{\text{catalyst}} \tag{11}$$

and

$$r_{\text{plasma}} = k_{+j}\prod_i C_i^{-c_{ij}} - k_{-j}\prod_i C_i^{c_{ij}} \tag{12}$$

The first term of Equation 11 represents the pressure difference between the inlet and outlet, the second one represents the contribution of bulk plasma reactions, and the third to surface catalytic reactions. $V(\text{m}^3)$ is the volume of the reactor, P_i(Pa) is the pressure of species i, t(s) is time, $v(\text{m}^3\,\text{s}^{-1})$ is flow rate, $P_{i,0}$ is the inlet pressure of P_i, $R(\text{m}^3\,\text{Pa}\,\text{K}^{-1}\,\text{mol}^{-1})$ is the gas constant, T(K) is bulk temperature, $r_{\text{plasma}}(\text{mol}\,\text{m}^{-3}\,\text{s}^{-1})$ is the rate of plasma reactions, $r_{\text{catalyst}}(\text{s}^{-1})$ is turnover frequency of an active site, as defined in Equation 8 and S(mol) is the number of active sites. $C_i(\text{mol}\,\text{m}^{-3})$ is the concentration of i and c_{ij} is the stoichiometric coefficient of species i in reaction j. At steady-state, $\frac{\partial P_i}{\partial t} = 0$. Equation 11 is solved with Equation 7 together to predict the pressures of gaseous species and coverages of surface species.

For both plasma and plasma-catalytic NO production, $v_{298\,\text{K},1\,\text{bar}} = 1.7\times10^{-6}\,\text{m}^3\,\text{s}^{-1}$, $P_{\text{N}_2,0} =$ 499.5 Pa, $P_{\text{O}_2,0} = 0.5$ Pa and $T = 873$ K. For plasma reactions only, we take the reactor vol-

ume to be consistent with the length of the heating mantle, where the length of the heating mantel = 30 cm, diameter of the reactor = 3.4 cm, $V = 2.7 \times 10^{-4}\,\mathrm{m}^3$ and $S = 0\,\mathrm{mol}$. For coupled plasma and catalysts, we take the volume of the porous Pt catalyst plug as the volume of the reactor, where the thickness of the Pt plug = 14 µm, diameter of the Pt plug = 2.5 cm, $V = 6.9 \times 10^{-9}\,\mathrm{m}^3$ and $S = 2.3 \times 10^{-7}\,\mathrm{mol}$.

Supplementary Fig. 8 shows the total results of Fig.4, including the outlet partial pressures of N, O and O_2 in both integral reactor models of plasma reactions and plasma catalytic reactions.

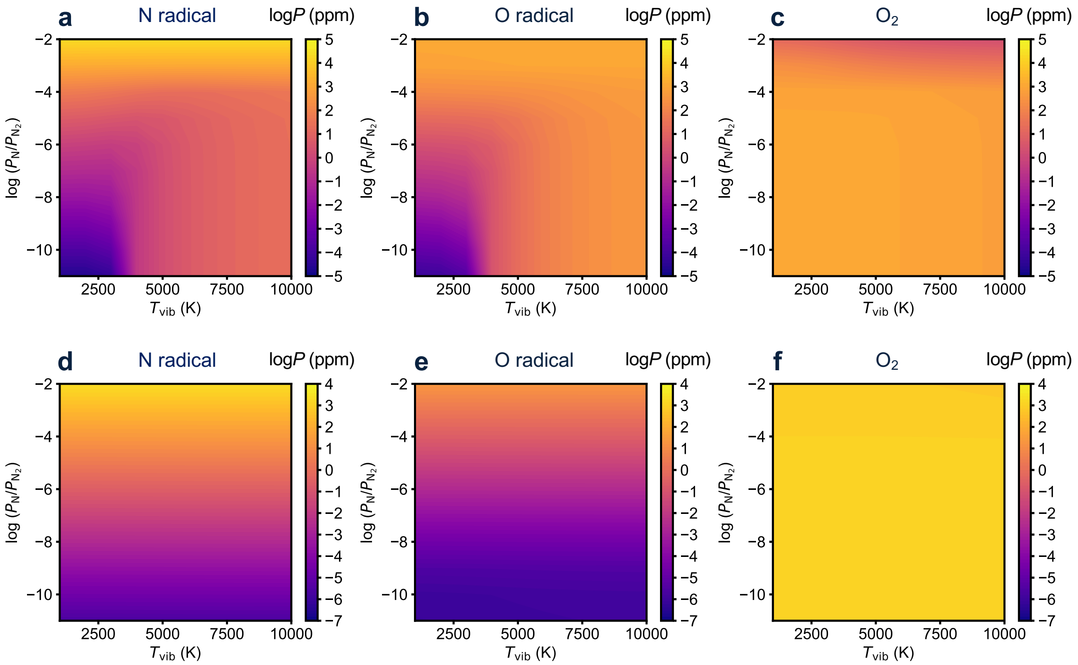

Supplementary Figure 8: Outlet pressures (ppm) of (a) N, (b) O and (c) O_2 of plasma reactions (Fig. 4a) against T_{vib} and P_{N}. Outlet pressures (ppm) of (d) N, (e) O and (f) O_2 of plasma catalytic reactions (Fig. 4b) against T_{vib} and P_{N}.

Supplementary Fig. 9 shows the comparison of NO concentrations from plasma and plasma catalytic reactors with $V_{\mathrm{plasma\,catalysis}}$ set as V_{plasma}. Here, the outlet NO concentrations are comparable in these two systems, suggesting the ratio between reactor volume and number of active sites determine the relative contributions of plasma phase and surface NO production. Test calculations show that the outlet composition converge with decreasing

volume of the plasma catalytic reactor. The $V_{\text{plasma catalysis}}$ used in Fig. 4 yields a converged outlet composition.

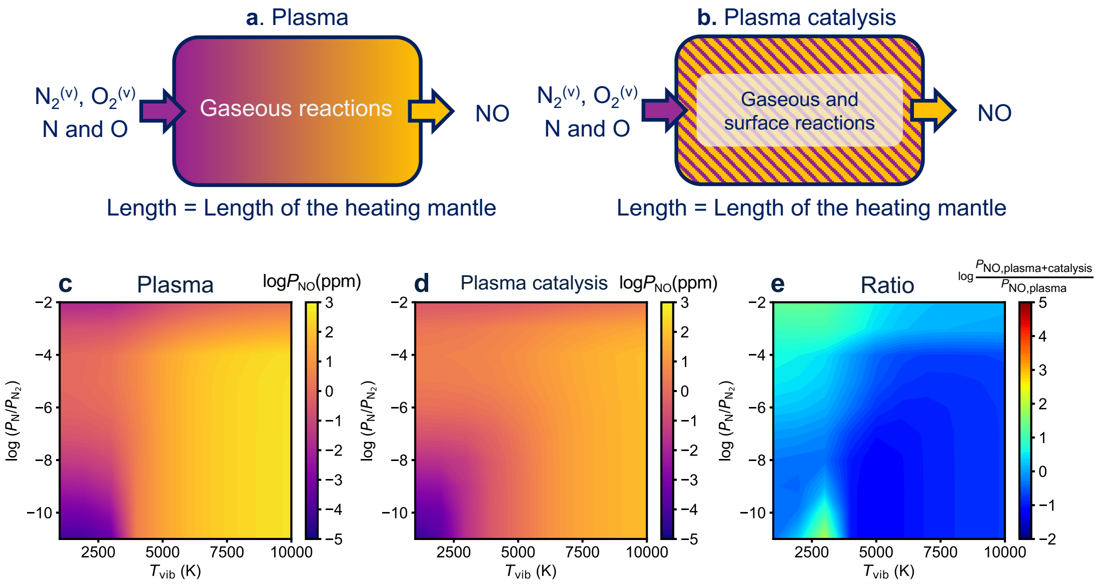

Supplementary Figure 9: Schematic representations of integral reactor models for (a) plasma reactions and (b) plasma catalytic reactions, respectively. The volume of (b) is artificially set to be the same as (a). Outlet NO pressures (ppm) of (c) plasma reactions and (b) plasma catalytic reactions against T_{vib} and P_{N}. (e) The ratio of NO produced in (c) and (d).

To understand the decreasing NO concentration at high N density in the plasma-only, non-catalytic reactor model, we plot NO concentration and the rate of each elementary reaction as a function of P_{N} at three representative vibrational temperatures in Supplementary Fig. 10. Supplementary Fig. 10a shows that NO concentration decreases sharply when P_{N} is larger than $10^{-5}P_{\text{N}_2}$. As shown in Supplementary Fig. 10b, the rates of both Zeldovich reactions keep constants when $P_{\text{N}} < 10^{-5}P_{\text{N}_2}$ and $T_{\text{vib}} = 3000\,\text{K}$. O_2+N↔NO+O contributes more to the NO rates than N_2+O↔NO+N. At high P_{N}, the rate of O_2+N↔NO+O increases while the rate of N_2+O↔NO+N decreases more substantially. The net NO production rate therefore deceases with P_{N} because of the high rate of NO reacting with N. Similar results are observed at higher T_{vib} (Supplementary Fig. 10c and d).

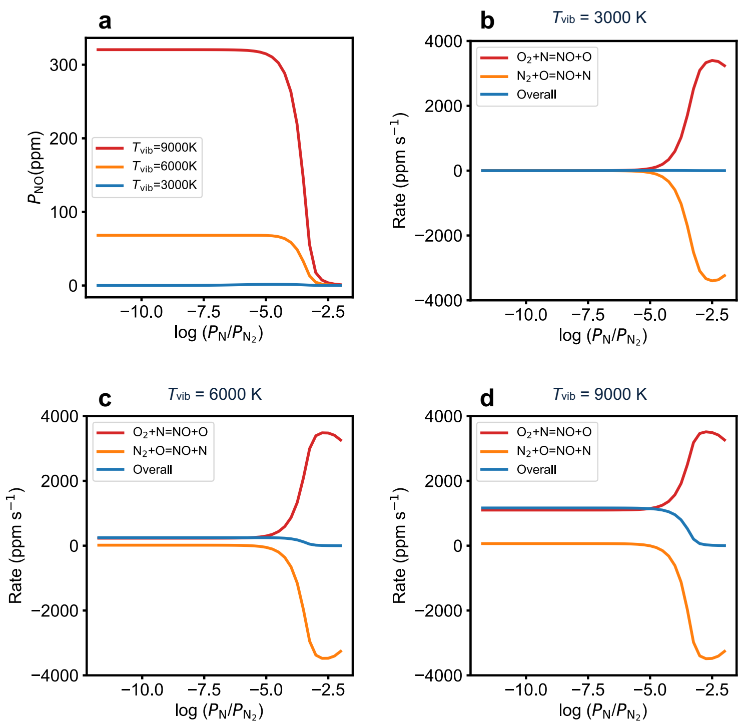

Supplementary Figure 10: Effect of N radicals on NO production. (a) NO production as a function of N-to-N_2 ratio. Net rates of Zeldovich reactions at vibrational temperature of (b) 3000 K, (c) 6000 K, (d) 9000 K.

Theoretical energy consumption

We estimate the theoretical energy consumption $E_{\text{consumption}}$(MJ/mol NO) at different plasma parametric conditions with the following equations:

$$E_{\text{consumption}} = (E_{\text{N}_2,\text{vib}} + E_{\text{N},\text{radical}} + E_{\text{O}_2,\text{vib}} + E_{\text{O},\text{radical}}) \times \frac{P_{\text{total}}}{P_{\text{NO}}} \tag{13}$$

$$E_{i,\text{vib}} = \sum_{v=0}^{10} p_{i,v} E_{i,v} \frac{P_i}{P_{\text{total}}} \tag{14}$$

and

$$E_{i,\text{radical}} = E_{i,\text{dissociation}} f_{i,\text{dissociation}} \frac{P_i}{P_{\text{total}}} \tag{15}$$

where $E_{\mathrm{i,vib}}$ is the energy consumption of vibrational excitation of i, $E_{\mathrm{i,radical}}$ is the energy required to dissociate a diatomic molecule i to radicals, P_{total} is the total pressure of N_2 and O_2, P_{NO} is the pressure of formed NO, $p_{i,v}$ is the normalized density of vibrational excited state v defined in Equation 4, $E_{i,v}$ is the energy of vibrational excited state relative to the ground state, $E_{i,\mathrm{dissociation}}$ is the bond-dissociation energy of molecule i and $f_{i,\mathrm{dissociation}}$ is the dissociation fraction of molecule i. We assume heat recovers and thus neglect heat energy in the analysis. Inclusion of energy to heat from 473 to 873 K increases the minimum energy to 42.5 $\mathrm{MJ/mol_{NO}}$.

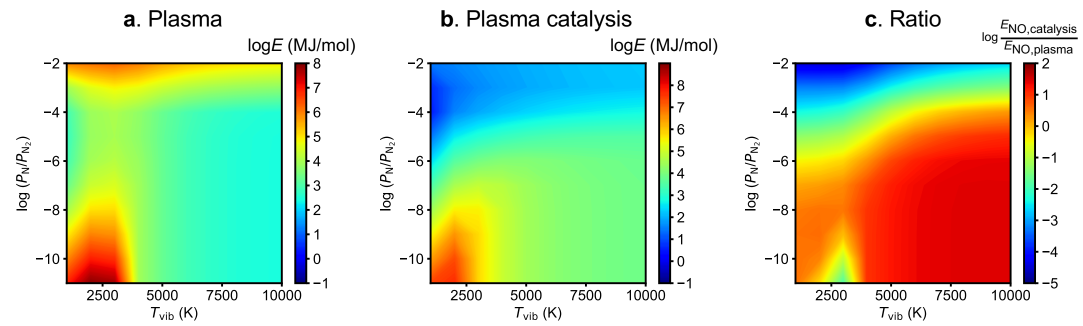

Supplementary Figure 11: Energy consumption, MJ/(mol NO) of (a) plasma reactions and (b) plasma catalytic reactions against T_{vib} and P_{N} at 873 K in integral reactors shown in Figure 3, where the inlet pressures of N_2 and O_2 are 4.995 and 0.005 bar, respectively. The energy consumption is calculated based on the activation of N_2-O_2 by plasma. (c) The ratio of energy consumptions in (a) and (b).

Intrinsic NO production and TOF over Pt(211) as a function of oxygen mole fractions

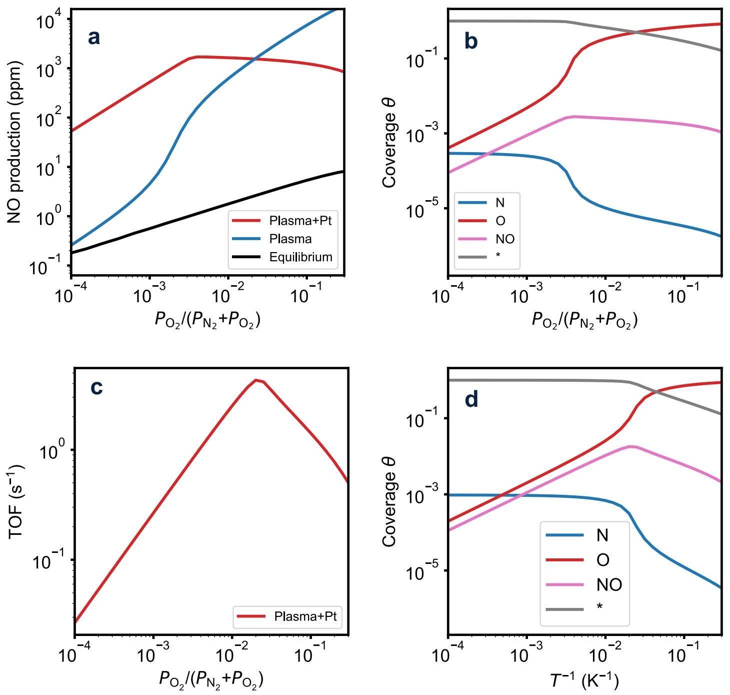

Supplementary Figure 12: (a) Microkinetic prediction of NO production as a function of O_2 pressure in both plasma and plasma catalytic systems with $P_N = 2 \times 10^{-3} P_{N_2}$ and $T_{vib} = 6000\,K$ at 873 K. The active Pt sites is 230 nmol in the plasma catalytic model. The length of the reactor for the plasma model equals to the length of the heating mantle and the reactor length equals to the thickness of the Pt film in the plasma catalytic model. (b) The surface coverages of Pt(211) in (a). (c) Turnover frequency as a function of O_2 pressure over Pt(211) with fixed pressures of reactants. The reactant pressures and reaction conditions are the same as the inlet pressures of (a). (d) The surface coverages of Pt(211) in (c).

Surface coverages of Pt(211) in the integral reactor series

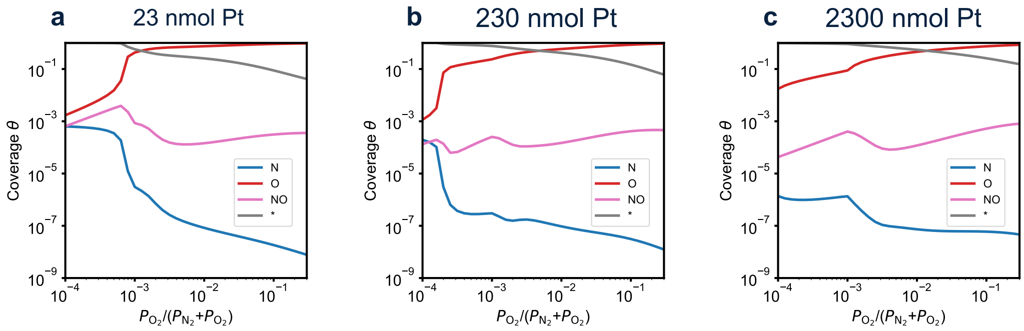

Supplementary Figure 13: The surface coverages of Pt(211) in microkinetic prediction of NO production as a function of O_2 pressure with the combination of three reactors (Figure 6). $P_N = 2 \times 10^{-3} P_{N_2}$ and $T_{vib} = 6000\,K$ at 873 K. The active Pt sites are (a) 23 nmol, (b) 230 nmol and (c) 2300 nmol in the plasma catalytic model.

NO production from a plug flow reactor model

We assumed the flow in the integral reactor model is well-mixed. Here we examine this assumption by comparing product concentrations with a series of CSTRs to represent a steady-state plug flow reactor (PFR), which captures the axial gradients of gas pressures. We replaced the single CSTR with a series of CSTRs at constant total volume. As shown in Supplementary Fig. 14a, plasma-only NO production converges at 10 CSTRs. We then plot the NO production of coupled plasma and Pt using 5 CSTRs to represent the pre- or post-catalyst regions. Supplementary Fig. 14b shows this model predicts the same NO concentrations at low O2 mole fractions and about 10% higher NO concentrations at intermediate and high O2 mole fractions than results in Fig. 6, where one CSTR was used to represent the pre- or post-catalyst regions.

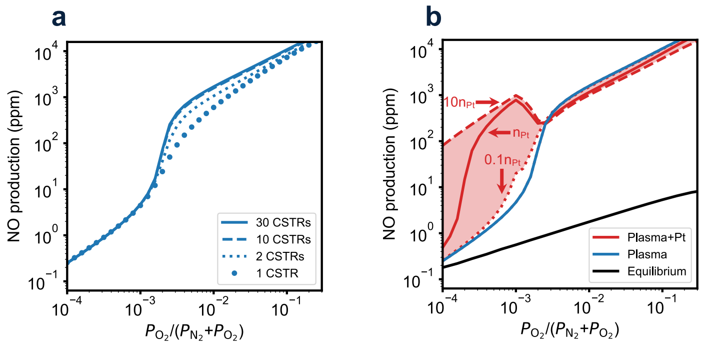

Supplementary Figure 14: (a) NO production as a function of O_2 pressure in the plasma integral reactor simulated using different numbers of identical CSTRs with the total volume fixed. (b) NO production as a function of O_2 pressure in both both plasma and plasma catalytic integral reactors simulated with 10 CSTRs for the plasma-only regions.

Semi-log plot of observed product concentrations

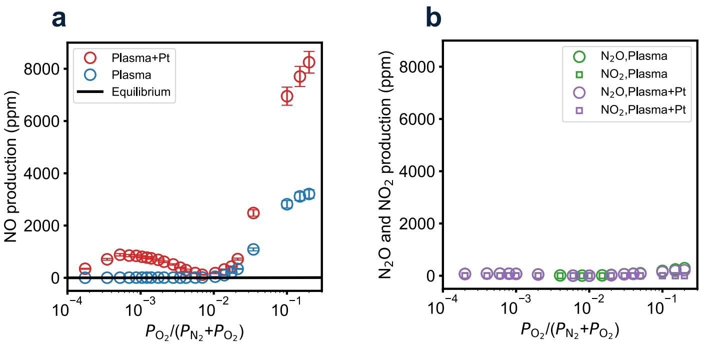

Supplementary Figure 15: Semi-log plot of observed (a) NO and (b) N_2O and NO_2 production as a function of O_2 pressure ratio. Total reactor pressure is 5 mbar.

Variation of vibrational temperature and radical densities at different oxygen mole fractions

Vibrational temperature: Vibrational temperature is observed to decrease with increasing O_2 mole fraction in previously reported low-pressure plasmas.[18,19] We assumed $T_{\rm vib}$ decreases linearly from 6000 to 5000 K when $\log_{10}\frac{P_{O_2}}{P_{O_2}+P_{N_2}}$ increases from -4 to -1. Supplementary Fig. 16a shows NO concentration decreases at high O_2 mole fractions. The overall

trends of NO concentrations are unvaried with decreasing T_{vib}.

Radical densities: $\frac{P_{\mathrm{N}}}{2P_{\mathrm{N_2}}}$ was reported to vary non-monotonically or decrease monotonically with O_2 mole fractions.[19–21] We test both dependencies here. We first assumed $\log_{10}\frac{P_{\mathrm{N}}}{2P_{\mathrm{N_2}}}$ increases linearly from -3 to -2.5 when $\log_{10}\frac{P_{\mathrm{O_2}}}{P_{\mathrm{O_2}}+P_{\mathrm{N_2}}}$ increases from -4 to -2. $\log_{10}\frac{P_{\mathrm{N}}}{2P_{\mathrm{N_2}}}$ then decreases from -2.5 to -3 when $\log_{10}\frac{P_{\mathrm{O_2}}}{P_{\mathrm{O_2}}+P_{\mathrm{N_2}}}$ increases from -2 to -1. Supplementary Fig. 16b shows that higher $\frac{P_{\mathrm{N}}}{P_{\mathrm{N_2}}}$ at intermediate O_2 mole fractions enhances NO production of coupled plasma and Pt while inhibits NO production of the plasma only reactor. Coupled plasma and catalyst therefore produces more NO than plasma in a wider range of O_2 mole fractions. Those trends better match the experimental observations.

We then assumed $\log_{10}\frac{P_{\mathrm{N}}}{2P_{\mathrm{N_2}}}$ decreases linearly with O_2 mole fractions from -3 to -4 when $\log_{10}\frac{P_{\mathrm{O_2}}}{P_{\mathrm{O_2}}+P_{\mathrm{N_2}}}$ increases from -4 to -1. At intermediate O_2 mole fractions, NO concentration increases for the plasma-only reactor because of less N inhibition. NO production from coupled plasma and Pt, however, decreases at intermediate O_2 mole fractions. Coupled plasma and catalyst produces more NO than plasma in a narrower range of O_2 mole fractions.

Above simulations assume $\frac{P_{\mathrm{O}}}{P_{\mathrm{O_2}}}$ is always $10\frac{P_{\mathrm{N}}}{P_{\mathrm{N_2}}}$. Simulations of N_2-O_2 flowing afterglows further show O_2 dissociation fraction remains almost constant at different O_2 mole fractions.[20,21] Supplementary Fig. 16d shows NO production with decreasing $\frac{P_{\mathrm{N}}}{2P_{\mathrm{N_2}}}$ and unvaried $\frac{P_{\mathrm{O}}}{2P_{\mathrm{O_2}}}$. NO concentrations increase at high O_2 mole fractions and the overall trends remain unchanged.

The dependence of vibrational temperature and radical densities on O_2 mole fraction is observed to vary across different plasmas.[18–20] Above results show uncertainties in the plasma characteristics do not change the conclusions. Our modeling approach is therefore robust. Largest sensitivities are to $\frac{P_{\mathrm{N}}}{P_{\mathrm{N_2}}}$ at intermediate O_2 fractions and T_{vib} at high O_2 fraction and variations of NO production are within one order of magnitude. Accurate estimation of the T_{vib} and P_{N} dependence on O_2 fractions could help better predict O_2 mole fractions at which coupled plasma and catalysts enhances NO production.

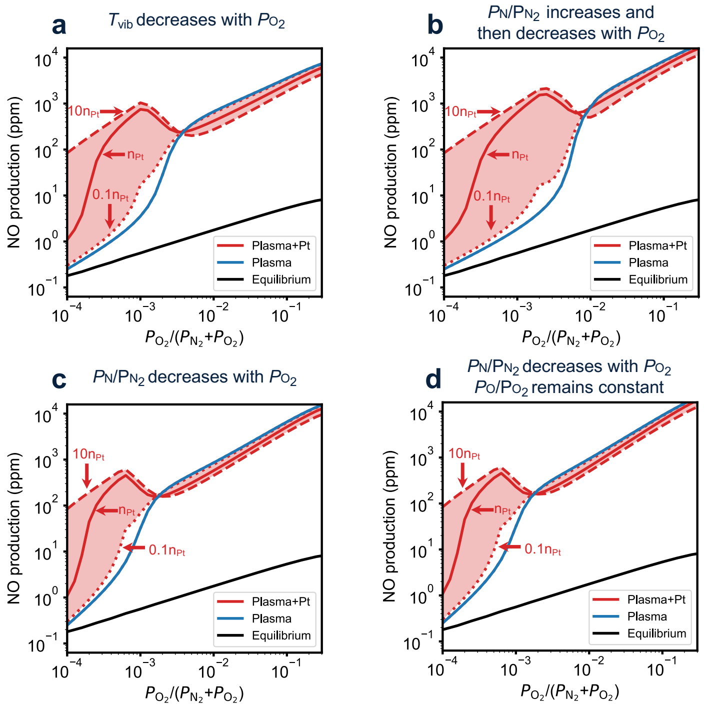

Supplementary Figure 16: NO production as a function of O_2 pressure in plasma-only and plasma-catalytic reactor series at bulk temperature 873 K. Red solid line represents a Pt active site number of 230 nmol and dashed and dotted line represent order-of-magnitude variations in that number. Total reactor pressure is 5 mbar. (a) $P_N = 2 \times 10^{-3} P_{N_2}$ and T_{vib} decreases linearly with $\log_{10} \frac{P_{O_2}}{P_{O_2}+P_{N_2}}$. (b) $T_{vib} = 6000$ K. $\log_{10} \frac{P_N}{2P_{N_2}}$ increases and decreases with $\log_{10} \frac{P_{O_2}}{P_{O_2}+P_{N_2}}$. (c) $T_{vib} = 6000$ K and $\log_{10} \frac{P_N}{2P_{N_2}}$ decreases linearly with $\log_{10} \frac{P_{O_2}}{P_{O_2}+P_{N_2}}$. (d) Same as (c) except that $\log_{10} \frac{P_O}{2P_{O_2}} = -2$ at all O_2 mole fractions.

Supplementary References

(1) Chase Jr, M. W. *NIST-JANAF Thermochemical Tables*; Journal of Physical and Chemical Reference Data Monograph No.9; National Institute of Standards and Technology: New York, 1998.

(2) Nørskov, J. K.; Studt, F.; Abild-Pedersen, F.; Bligaard, T. *Fundamental Concepts in Heterogeneous Catalysis*; John Wiley & Sons, 2014.

(3) Nørskov, J. K.; Bligaard, T.; Logadottir, A.; Bahn, S.; Hansen, L. B.; Bollinger, M.;

Bengaard, H.; Hammer, B.; Sljivancanin, Z.; Mavrikakis, M.; Xu, Y.; Dahl, S.; Jacobsen, C. J. Universality in heterogeneous catalysis. *J. Catal.* **2002**, *209*, 275–278.

(4) Ma, H.; Schneider, W. F. Structure- and Temperature-Dependence of Pt-Catalyzed Ammonia Oxidation Rates and Selectivities. *ACS Catal.* **2019**, *9*, 2407–2414.

(5) Bajpai, A.; Mehta, P.; Frey, K.; Lehmer, A. M.; Schneider, W. F. Benchmark First-Principles Calculations of Adsorbate Free Energies. *ACS Catal.* **2018**, *8*, 1945–1954.

(6) Falsig, H.; Shen, J.; Khan, T. S.; Guo, W.; Jones, G.; Dahl, S.; Bligaard, T. On the Structure Sensitivity of Direct NO Decomposition over Low-Index Transition Metal Facets. *Top. Catal.* **2014**, *57*, 80–88.

(7) Cortright, R.; Dumesic, J. Kinetics of heterogeneous catalytic reactions: Analysis of reaction schemes. *Adv. Catal.* **2001**, *46*, 161–264.

(8) Dumesic, J. *The Microkinetics of Heterogeneous Catalysis*; ACS Professional Reference Book; American Chemical Society: Washington, 1993; pp 23–54.

(9) Treanor, C. E.; Rich, J. W.; Rehm, R. G. Vibrational Relaxation of Anharmonic Oscillators with Exchange-Dominated Collisions. *J. Chem. Phys.* **1968**, *48*, 1798–1807.

(10) Fridman, A. *Plasma chemistry*; Cambridge university press, 2008.

(11) Irikura, K. K. Experimental Vibrational Zero-Point Energies: Diatomic Molecules. *J. Phys. Chem. Ref. Data* **2007**, *36*, 389–397.

(12) Mehta, P.; Barboun, P.; Herrera, F. A.; Kim, J.; Rumbach, P.; Go, D. B.; Hicks, J. C.; Schneider, W. F. Overcoming ammonia synthesis scaling relations with plasma-enabled catalysis. *Nat. Catal.* **2018**, *1*, 269–275.

(13) Grabow, L. C. In *Computational Catalysis*; Asthagiri, A., Janik, M. J., Eds.; Royal Society of Chemistry: London, 2013; pp 1–58.

(14) Campbell, C. T. The Degree of Rate Control: A Powerful Tool for Catalysis Research. *ACS Catal.* **2017**, *7*, 2770–2779.

(15) Capitelli, M.; Ferreira, C. M.; Gordiets, B. F.; Osipov, A. I. *Plasma kinetics in atmospheric gases*; Springer-Verlag Berlin Heidelberg, 2000; Vol. 31.

(16) Wray, K. L.; Teare, J. D. Shock-Tube Study of the Kinetics of Nitric Oxide at High Temperatures. *J. Chem. Phys.* **1962**, *36*, 2582–2596.

(17) Michael, J. V.; Lim, K. P. Rate constants for the N_2O reaction system: Thermal decomposition of N_2O; $N+NO \rightarrow N_2+O$; and implications for $O+N_2 \rightarrow NO+N$. *J. Chem. Phys.* **1992**, *97*, 3228–3234.

(18) Tan, H.; Nezu, A.; Matsuura, H.; Akatsuka, H. Spectroscopic determination of vibrational and rotational temperatures of NO molecules in N_2–O_2 mixture microwave discharge. *Jpn. J. Appl. Phys.* **2014**, *54*, 01AB06.

(19) Nahomy, J.; Ferreira, C. M.; Gordiets, B.; Pagnon, D.; Touzeau, M.; Vialle, M. Experimental and theoretical investigation of a N_2-O_2 DC flowing glow discharge. *J. Phys. D: Appl. Phys.* **1995**, *28*, 738–747.

(20) Pintassilgo, C. D.; Loureiro, J.; Guerra, V. Modelling of a N_2–O_2 flowing afterglow for plasma sterilization. *J. Phys. D: Appl. Phys.* **2005**, *38*, 417–430.

(21) Kutasi, K.; Pintassilgo, C. D.; Loureiro, J.; Coelho, P. J. Active species in a large volume N_2–O_2 post-discharge reactor. *J. Phys. D: Appl. Phys.* **2007**, *40*, 1990–2001.
